# The interactive effect between economic uncertainty and life history strategy on corrupt intentions: a life history theory approach

**DOI:** 10.3389/fpsyg.2024.1361158

**Published:** 2024-04-30

**Authors:** Xueying Sai, Lei Zhu

**Affiliations:** Department of Psychology, Fudan University, Shanghai, China

**Keywords:** economic uncertainty, life history strategy, desire for power, corrupt intentions, evolutionary psychology perspective

## Abstract

**Introduction:**

Why do some people show more corruption when facing uncertain environment? The present study aimed to give a plausible answer from an evolutionary perspective: this might be rooted in people’s different life history strategies (slow vs. fast).

**Methods:**

The present study measured the participants’ corrupt intentions by a hypothetical scenario and primed the feeling of economic environmental uncertainty by requiring the participants to read economic uncertainty (vs. neutral) materials.

**Results:**

It is revealed that the participants with fast life history strategies had stronger corrupt intentions after reading materials about economic uncertainty than reading neutral materials. In addition, the desire for power mediated the interactive effect between life history strategy and economic uncertainty on corrupt intentions for fast life history strategists.

**Discussion:**

This finding was discussed for its theoretical and practical implications from the perspective of life history theory.

## Introduction

Although sociologists have systematically investigated the negative effects of corruption on political and economic development (Dreher and Herzfeld, 2005; Grabova, 2014; Alfada, 2019; Mauro et al., 2019), only a few studies have been done to explore what factors influence individuals’ corruption. The present study aimed to reveal these influencing factors from an evolutionary perspective.

### Corruption

Corruption is a global problem that has existed across various cultures for centuries and is typically defined as the misuse of public power for private gain (Klitgaard, 1968; Shleifer and Vishny, 1993; Svensson, 2005). Corruption disrupts the allocation of public spending (Mustapha, 2014), undermines social justice (Rothstein and Uslaner, 2005), and threatens democracy and morality (Bai et al., 2014). The widespread negative effects of corruption on society have led to various studies on it in economics, sociology, and psychology.

Sociologists measure corruption at the national level by assessing citizens’ self-reported perceptions and experiences of corruption, such as Transparency International’s Annual Corruption Perception Index (CPI), which measures expert and public opinion on corruption across more than 150 countries. These datasets on cross-country perceptions of corruption provide important macro-level estimates of the impact of corruption on economic growth, social development, and societal well-being (Wei, 2000; Rothstein and Uslaner, 2005; Sulemana et al., 2017). However, economists and psychologists measure individual corrupt behaviors in the laboratory by corruption games, such as the three-person auction game (Köbis et al., 2017) and the bribery game (AIR) developed by Abbink et al. (2002). To measure individual corrupt behaviors, these games require participants to act as bribers, in which they can either bribe or not bribe various amounts of money. For further ecological validity, psychologists employ realistic hypothetical scenarios to capture participants’ corrupt intentions and behaviors. For instance, they presented a hypothetical scenario (Li et al., 2006; Tan et al., 2017) or required the participants to read a vignette (Torfason et al., 2013), and asked them whether they would accept a financial reward if they were in the same situation, or to what extent they agreed with the briber’s actions.

The above measurements allow researchers to explore corruption and identify various influencing factors, such as power (Wang and Sun, 2016), social norms (Köbis et al., 2015), and belief in a just world (Tan et al., 2016a). All these studies implied that corruption occurred when people in the positions of power or authority abused their influence to violate moral norms for their own benefit (Dungan et al., 2014). In other words, moral costs played a role during corruption-related activities (Banerjee, 2016), suggesting that corruption might be driven by the powerful group-based moral concerns (Dungan et al., 2014). Furthermore, some research aimed to reduce corruption by enhancing individuals’ sense of morality. For instance, DeCelles et al. (2012) found that power led individuals with a high moral identity to decrease corrupt behaviors, whereas it increased corruption among those with a low moral identity. These studies collectively suggested that corruption constituted immoral behaviors. However, previous studies mainly examined the relationship between morality and corruption cross-sectionally by relating individuals’ present immorality to their current corrupt intentions and behaviors but not examine the fundamental reasons for corruption from a developmental and evolutionary perspective.

### Life history theory and sociomoral mental processes and behaviors

An evolutionary explanation for such sociomoral mental processes and behaviors might be that (Zhu et al., 2018, 2019), in certain circumstances, individuals prefer personal present rewards (i.e., self-centeredness moral process) over future rewards like reputation and social support by considering others’ fitness (i.e., other-centeredness moral process). This prioritization of one’s own fitness over others’ is seen as adaptive in evolutionary terms as it provides immediate benefits.

Such a trade-off between present and future fitness is not specific to sociomoral mental processes and behaviors. Rather, it can be found in a variety of psychological processes and behaviors. According to life history (LH) theory, due to the inherently limited nature of resources and energy, all the individuals face difficult trade-offs between the acquisition of embodied capital for survival (e.g., knowledge, skills) and the acquisition of intrasexual competition for delivery genes (e.g., mating, parenting). Different decisions toward the trade-off might be influenced by two fundamentally different life courses that species use, which vary from slow to fast on a continuum (Figueredo et al., 2004, 2006; Bielby et al., 2007; Ellis et al., 2009; Nettle, 2010; Griskevicius et al., 2011a).

Specifically, individuals with a slow life history strategy tend to postpone marriage (Figueredo et al., 2004), have high group cohesion (Figueredo et al., 2005), and are more ethical (Gladden and Cleator, 2018). This strategy is associated with other-centered sociomoral mental processes and behaviors, with a tendency to prioritize others’ welfare even at personal costs (Zhu et al., 2018), and thus may not involve corruption. In contrast, individuals with a fast life history strategy are associated with anti-social behaviors (Figueredo et al., 2004), alcohol abuse (Figueredo et al., 2006), and positively related to the rates of murder, robbery, and assault (Dunkel et al., 2013). This strategy is associated with self-centered sociomoral mental processes and behaviors, with a preference for present self-interest (Zhu et al., 2018), and thus may involve corruption for private gain.

### Life history theory and environmental sensitivity

In addition, since life history strategies may result from physiological stress response systems tracking and internalizing early-life experiences of stress and danger, the differences in life history strategies can be observed in how individuals cope with unpredictable environmental threats (Boyce and Ellis, 2005; Del Giudice et al., 2011; Ellis et al., 2017; Ellis and Del Giudice, 2019). The Adaptive Calibration Model (ACM, Del Giudice et al., 2011) identifies four different patterns (i.e., Sensitive, Buffered, Vigilant, Unemotional) of stress responses corresponding to experiences in extremely low-stress to extremely high-stress environments (Del Giudice et al., 2011; Ellis et al., 2017; Ellis and Del Giudice, 2019). Responses to early-life low to moderate stress experiences include sensitive (i.e., upregulation) and buffered (i.e., downregulation) patterns, which might be considered as slow life history strategies. Conversely, responses to early-life high stress experiences include vigilant (i.e., upregulation) or unemotional (i.e., downregulation) patterns, which might reflect fast life history strategies. Thus, repeated exposures to safe and stable environments lead individuals to hold slow life history strategies (Figueredo et al., 2004), whereas repeated exposures to harsh and unpredictable environments lead individuals to hold fast life history strategies (White et al., 2013; Mittal and Griskevicius, 2014).

Further, the biological sensitivity to context model (BSC, Boyce and Ellis, 2005) suggests that the stress response system has developmental plasticity in that it monitors specific features of childhood circumstances and uses them as a basis for calibrating the stress response system during development to adaptively match the environments in one’s later life. The sensitization model discusses this in detail (Griskevicius et al., 2011b; Hill et al., 2013; White et al., 2013; Mittal and Griskevicius, 2014). It specifically describes how different life-history strategies calibrate their behaviors in facing uncertain and harsh environments later in life. Early exposures to unpredictable and harsh environments may “sensitize” life history strategies, shunting individuals toward distinct development paths that are either slow or fast (Mucignat Caretta et al., 1995). Such life history strategies adapted from early-life experiences ought to affect how individuals respond to some environment later in life.

And, the psychological differences associated with two life history strategies might lay “dormant” in benign ecological environments but might become active under uncertain environments (Griskevicius et al., 2011b; Hill et al., 2013; White et al., 2013; Mittal and Griskevicius, 2014). For example, reading news about economic uncertainty led individuals with a fast strategy, rather than individuals with a slow-life-history strategy, to tend to prefer immediate returns (Griskevicius et al., 2011b; Hill et al., 2013; White et al., 2013; Mittal and Griskevicius, 2014). Corruption is immoral behaviors that focus on short-term self-interest but impairs long-term collective interest (Li et al., 2021). Thus, we hypothesized that variations in life history strategy might explain the individual differences in corruption. Specifically, individuals with a fast life history strategy would have more corrupt intentions in the context of economic uncertainty.

### The possible mediating effect of the desire for power

In sum, from an evolutionary perspective, individuals might develop various adaptive strategies in response to uncertain environments to increase their chances of getting more resources such as power (Chen, 2018). According to Resource Control (RC) theory (Charlesworth, 1996), there are two kinds of resource competing strategies (coercive vs. prosocial). Prosocial strategies acquire resources through skills expertise and positive relationships with others, whereas coercive strategies acquire resources through stealing, deception, and violence. Chen (2017) revealed that individuals’ life history strategies partly determined which resource competing strategy they would use. As mentioned above, the reason why individuals with a fast life history strategy have more norm-violation behaviors such as anti-social behaviors (Figueredo et al., 2004) and murder, robbery or assault (Dunkel et al., 2013) might be that they are inclined to obtain more resources by inappropriate coercive strategies. Xu (2021) supported this idea by demonstrating that fast life history strategists were more likely to use coercive strategies when competing for resources with others. Similarly, corruption is also a norm-violation coercive strategy that fast life history strategists might use in order to acquire more resources such as power.

On the other hand, power is the ability to influence others through the control of resources (Galinsky et al., 2003; Keltner et al., 2003) and desire for power is the desire for such resources to control others. Thus, referring to RC theory, the reason why individuals with a fast life history strategy would have more corrupt intentions in the context of economic uncertainty might be that they are inclined to obtain more power resources (i.e., more desire for power). It is hypothesized that the desire for power would mediate the relationship between life history strategy, environmental uncertainty and corruption as an underlying mechanism. Individuals with fast life history strategies would have more desire for power and thus adopt coercive strategies such as corruption when facing uncertain environments. In contrast, individuals with slow life history strategies would have less desire for power and thus adopt prosocial strategies such as less corruption to maintain high socio-economic status and positive social relationships.

### The current research

In the current research, we explored the interactive effects between economic environmental uncertainty and life history strategy on corrupt intentions. We first measured the participants’ life history strategies and then adopted a sensitization model paradigm to manipulate the feeling of economic environmental uncertainty by requiring the participants to read economic uncertainty or neutral materials. Finally, the participants’ desire for power and corrupt intentions were measured. It should be noted that to increase ecological validity, a single-trial hypothetical scenario was used to capture the participants’ corrupt intentions for several reasons. First, the questionnaires which have inherent self-reflection bias may not be precise. Second, although the economic corruption games involve multiple trials, they do not exclusively measure corruption and also involve other psychological processes such as reciprocal cooperation. Also, repeated responses to the same experimental scenario might make the participants be aware of the research aim and confound the results. Thus, we directly measured the corrupt intentions by asking the participants to what extent they would accept financial rewards in a single-trial hypothetical scenario.

It is hypothesized that the interactive effects between economic environmental uncertainty and life history strategy on the desire for power and corrupt intentions would be observed in the way that the participants with fast life history strategies would have more desire for power and corrupt intentions when facing economic uncertainty. Also, the desire for power would mediate such interactive effects on corrupt intentions.

## Methods

### Participants

The sample size was determined by *a priori* power analysis using G∗Power 3.1 (Faul et al., 2007), focusing on the interaction of GLM with an estimated power of 0.95, and an effect size of Cohen’s *f* = 0.28 (Borenstein et al., 1990). The analysis determined that a sample size of 168 participants was required. We finally recruited 168 participants (115 men, 53 women) from the university community. The mean age of participants was 19.12 (*SD* = 1.22) years old. The participants were randomly assigned to an economic uncertainty (*N* = 84, *M* age = 19.18 years old) or a control group (*N* = 84, *M* age = 19.06 years old). All the participants were provided informed consent at the beginning and were debriefed and thanked in the end. This study was approved by the University Committee on Human Research Protection and was carried out in accordance with the approved guidelines. We report how we determined our sample size, all data exclusions (if any), all manipulations, and all measures in each study.

### Design and procedures

The participants were required to complete a memory task. First, before the memory test, they were required to answer some questions about themselves (i.e., complete the life history strategy questionnaire). Then, they were required to complete the memory test. For each group, 10 slides were presented and the participants were informed that they would be required to recognize specific details from the slides a few minutes later. Actually, the slides were used to manipulate the feeling of economic uncertainty. There was one start slide, one conclusion slide and eight image slides. The start and conclusion slides presented a description and eight image slides included an image and a corresponding description. The participants could click to view the next slide. In order to let the participants have enough time to process the materials, the start and conclusion slides were presented for at least 3 s. Within 3 s, the participants could not click to view the next one. The image slides were presented for at least 10 s.

To prime the feeling of economic uncertainty, for the economics uncertain group, the slides presented news about economic uncertainty. The news materials were revised from a set of materials repeatedly used in previous studies (Griskevicius et al., 2013; Hill et al., 2013; Mittal and Griskevicius, 2014), which could successfully prime the feeling of economic uncertainty. We adapted it with more understandable images for undergraduate participants, including the stressful employment situation, excessive-priced housing, declining purchasing power, etc. For the image slides, a brief description accompanied each image (e.g., “In 2021, there would be 9.09 million college graduate students, with an increase of 350,000 year-by-year, and the number of people seeking employment is extremely increasing”). In the start and conclusion slides, a statement claiming that these trends reflected the uncertainty and unstable of the future economic environments, which would be challenging and stressful was presented without images. For the control group, refer to Griskevicius et al. (2013), we used images depicting objects found in an office, such as staplers, paper clips, and binders, accompanied with a brief description of the objects in each image for the image slides. In the start and conclusion slides, a statement claiming that there were a lot of office supplies on the desk was presented without images.

After the slide presentation, the participants were required to answer some questions to allow for “memory decay.” First, to check whether the experimental manipulation was valid, the participants were required to rate to what extent they perceived the current economic environment was uncertain. They were required to report their agreements on a 6-point scale (1 = strongly disagree; 6 = strongly agree) about 3 statements (i.e., “today’s survival pressure is increasing,” “today’s social development is more unpredictable than before” and “today’s economic development is more unstable than before”). The ratings were summed to compute the environment uncertainty index (*α* = 0.85). Then, the participants were required to complete the desire for power questionnaire and the student council corruption scenario task. Finally, they were required to complete a recognition test with two images (one old image presented in the slides and one new image which had not been presented before).

### Measures

#### Life history strategy

The life history strategies were measured by the Chinese version of the Mini-K (Sai et al., 2022). The participants were required to report their agreements on a 6-point scale (1 = strongly disagree; 6 = strongly agree) about 19 statements (e.g., “I often make plans in advance.”). The ratings were summed to compute the life history strategy index with higher scores indicating a slower life history strategy (*α* = 0.79).

#### Desire for power

The desire for power was measured by a four-item questionnaire (*cf.* Lammers et al., 2016; e.g., “I would like to have more power”). The participants were required to report their agreements on a 6-point scale (1 = strongly disagree; 6 = strongly agree) about the four statements. The ratings were summed to compute the desire for power index with higher scores indicating more desire for power (*α* = 0.83).

#### Corrupt intention

The corrupt intentions were measured by a student council corruption scenario task (Li et al., 2006; Tan et al., 2017). The scenario depicted 50 candidates competing for five scholarships offered by an international charitable organization. These candidates hoped that the volunteer association’s president would provide them with a fake volunteer service certificate at the cost of fairness. This would help them succeed in obtaining the funding. After reading the scenario, participants were required to imagine they were the volunteer association’s president and entitled to provide the volunteer service certification. Then, they were required to answer one question about their corrupt intention (i.e., “Now, there is a candidate who offers you 5,000 RMB and hopes that you can provide the faked certificate, to what extent you will accept the money and give him the faked certificate”). Participants were required to give a rating on a scale from 0 to 100, with 0 indicating complete disagreement to provide the fake certifications and 100 indicating complete agreement.

## Results

### Manipulation check

An independent samples *t*-test confirmed the experimental manipulation was effective, *t*(166) = 2.18, *p* = 0.031, *d* = 0.16, 95% CI [−2.02, −0.10]. The economic uncertain group (*M* = 12.77, *SD* = 3.44) perceived the current economic environment to be more uncertain and unpredictable than the control group (*M* = 13.83, *SD* = 2.84).

### Desire for power

First, to test how economic uncertainty and life history strategy affected the desire for power, a general linear model (GLM) analysis was carried out, with economic uncertainty as the fixed factor, life history strategy as the covariate, and the desire for power as the dependent variable. As our prediction, the interaction between economic uncertainty and life history strategy was significant, *F*(1,164) = 4.52, *p* = 0.035, *η_p_^2^* = 0.03. Besides, there were no significant main effects of economic uncertainty or life history strategy, *Fs* < 2.34, *ps* > 0.13.

**Figure 1:**
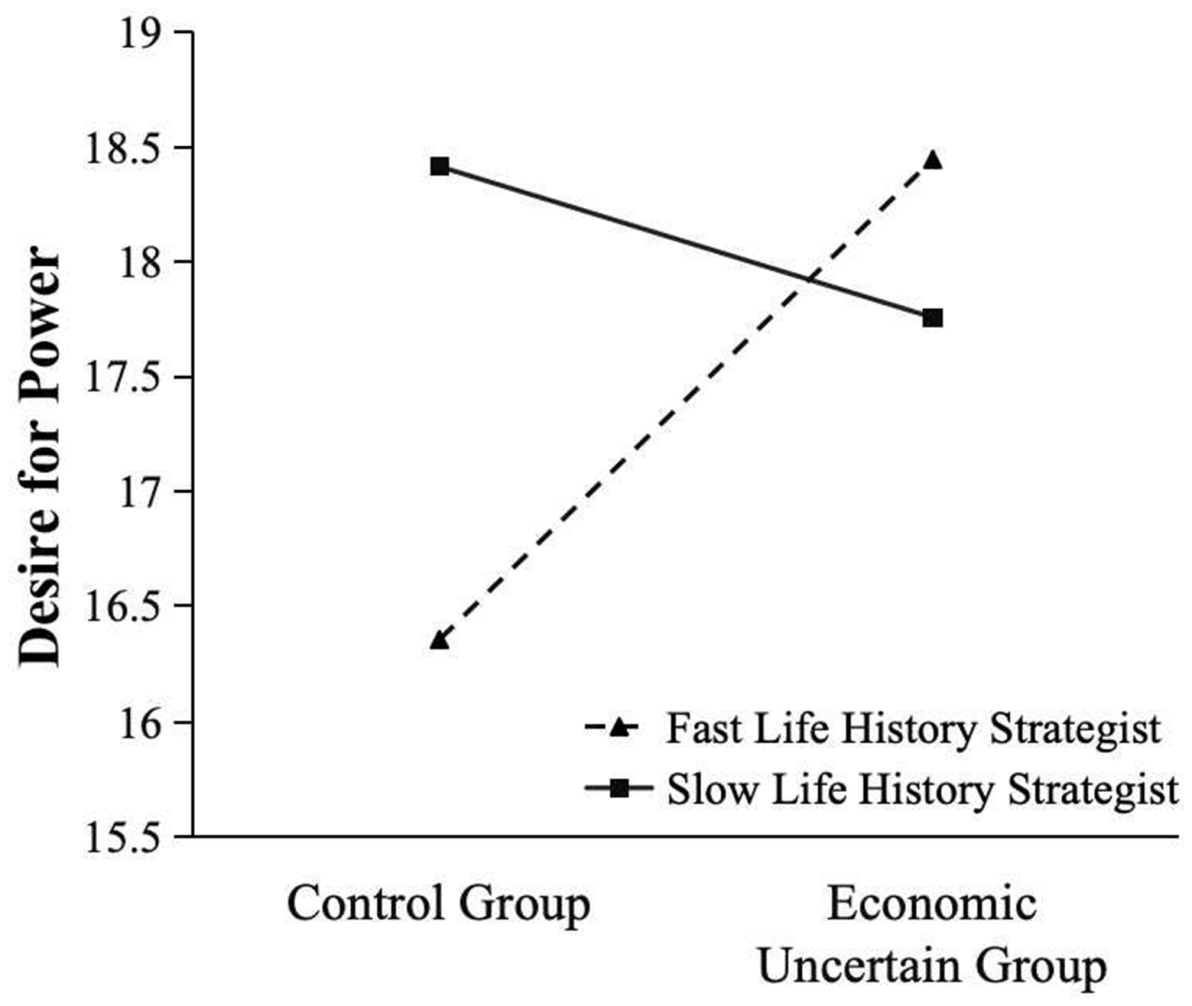
The interactive effect between life history strategy and economic uncertainty on desire for power.

Next, as shown in Figure 1, the simple slope analysis revealed that the participants with fast life history strategies (1 *SD* below the scale mean) had more desire for power after reading materials about economic uncertainty than reading control materials, *t*(164) = 2.31, *p* = 0.022. However, the participants with slow life history strategies (1 *SD* above the scale mean) had less desire for power after reading materials about economic uncertainty than reading control materials, *t*(164) = 0.73, *p* = 0.466.

Finally, two regression analyses were carried out separately for two groups. For the control group, the participants with a slow life history strategy had more desire for power than those with a fast life history strategy, *β* = 0.29, *p =* 0.008. However, for the economic uncertain group, the participants’ life history strategy was not significantly related to their desire for power, *β* = −0.07, *p* = 0.522.

### Corrupt intentions

Similarly, a GLM analysis on corrupt intentions with economic uncertainty as the fixed factor, and life history strategy as the covariate revealed that the main effect of life history strategy was significant, with the faster life history strategists reporting more corrupt intentions, *F*(1,164) = 13.33, *p* < 0.001, *η_p_^2^* = 0.08. In addition, although the main effect of economic uncertainty was not significant, the interaction between economic uncertainty and life history strategy was also significant, *F*(1,164) = 5.73, *p* = 0.018, *η_p_^2^* = 0.04.

**Figure 2:**
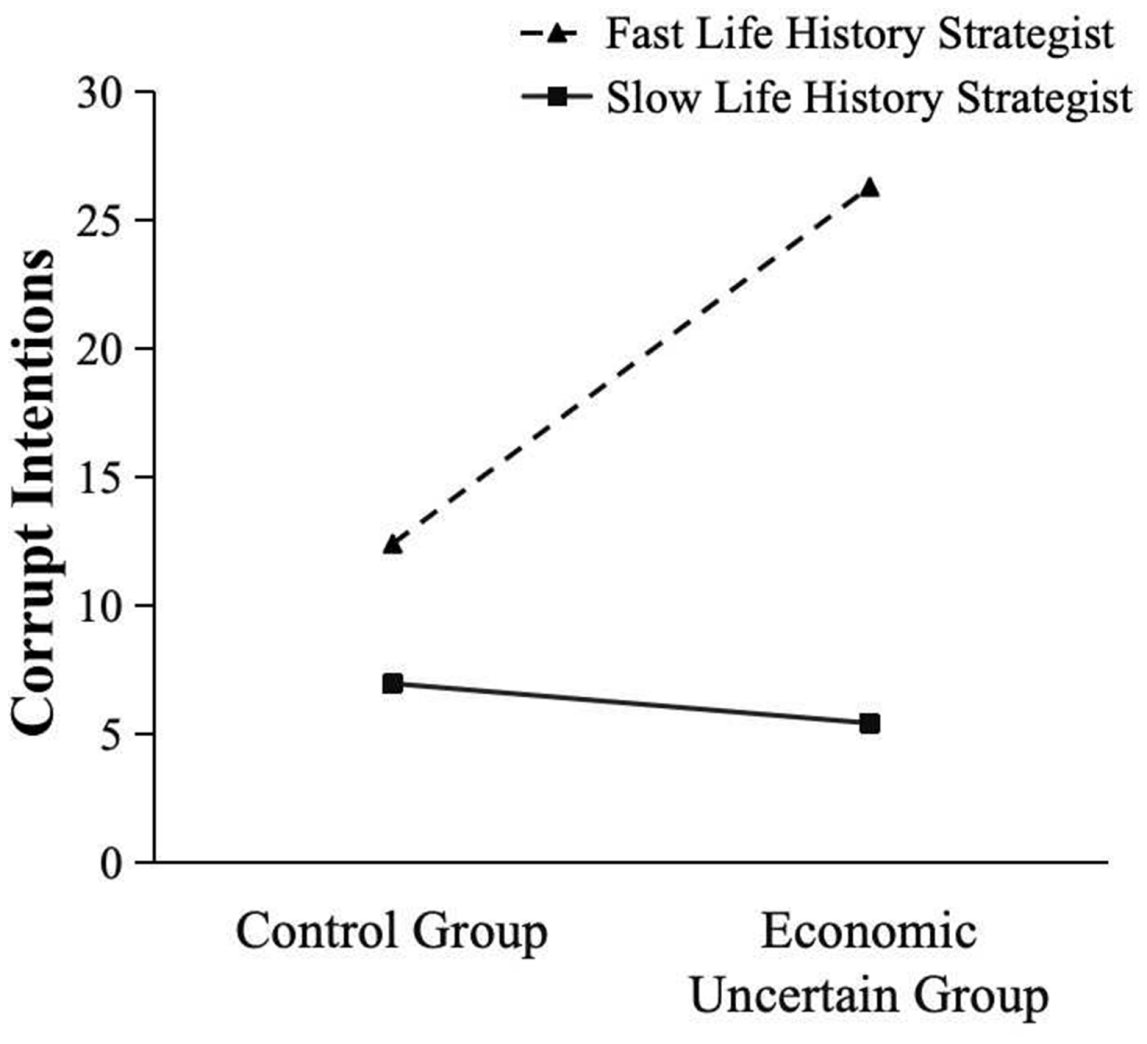
The interactive effect between life history strategy and economic uncertainty on corrupt intentions.

As shown in Figure 2, the simple slope analysis revealed that the participants with fast life history strategies (1 *SD* below the scale mean) had stronger corrupt intentions after reading materials about economic uncertainty than reading control materials, *t*(164) = 3.08, *p* = 0.003. The participants with slow life history strategies (1 *SD* above the scale mean) had less corrupt intentions after reading materials about economic uncertainty than reading control materials, *t*(164) = 0.34, *p* = 0.733.

Finally, two regression analyses were carried out separately for two groups. For the control group, consistent with the sensitization model, the corrupt intentions did not significantly differ between the participants with different life history strategies, *β* = −0.20, *p* = 0.089. However, for the economic uncertain group, the participants with a fast life history strategy had stronger corrupt intentions than those with a slow life history strategy, *β* = −0.37, *p =* 0.001.

### Moderated mediation analyses

**Figure 3:**
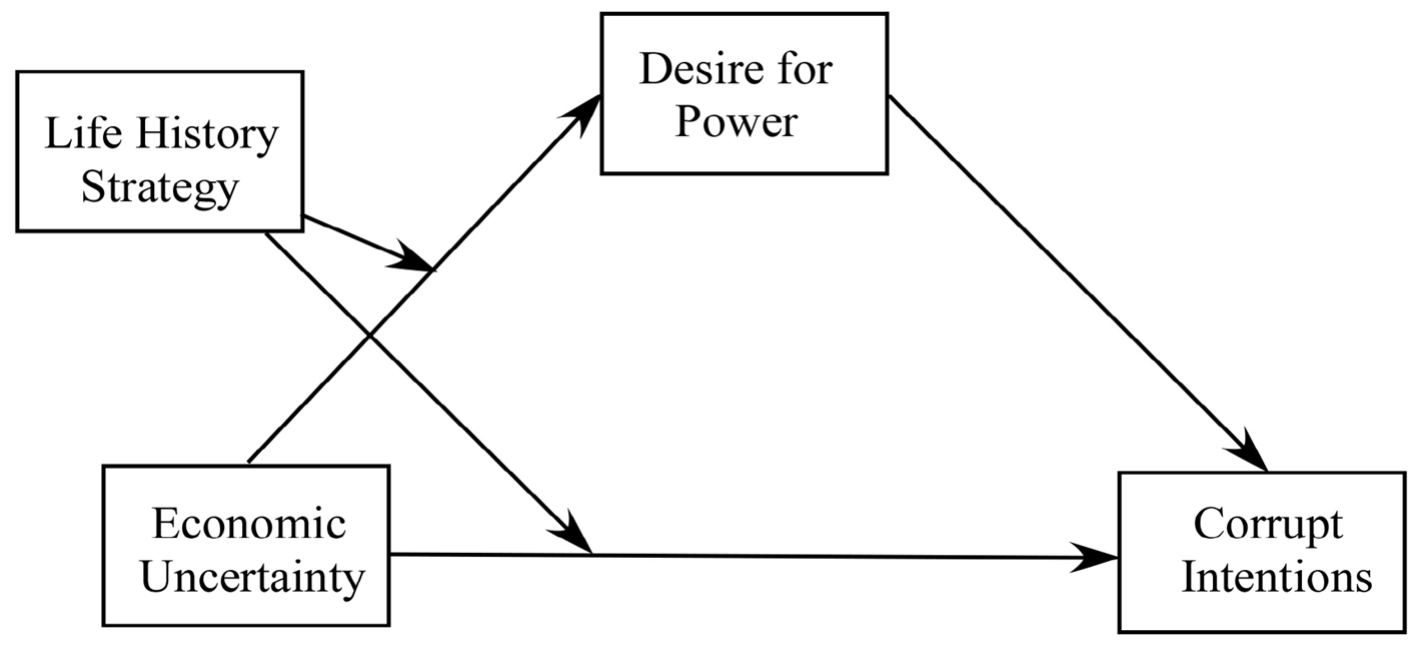
The hypothesized moderated mediation model.

A moderated mediation model following Hayes (2021, Model 8) with the routes from economic uncertainty (independent variable) to corrupt intention (dependent variable) and from economic uncertainty (independent variable) to the desire for power (mediating variable) both moderated by the life history strategy (moderating variable) was adopted (Figure 3). Consistent with the results of GLM, the interactive effect between economic uncertainty and life history strategy was significant on both the desire for power, *β* = −0.40, *z =* 6.52, *p* < 0.001, and corrupt intentions, *β* = −0.40, *z =* −5.52, *p* < 0.001. Moreover, the desire for power significantly predicted corrupt intentions, *β* = 0.21, *p =* 0.010.

Most importantly, consistent with our prediction, a 5,000-resample bootstrap revealed that for the participants with fast life history strategies (1 *SD* below the scale mean), the direct effects of economic uncertainty and life history strategy interaction [*β* = 0.51, *z =* 3.50, *p* < 0.001, 95% CI (5.20, 18.43)] and indirect effects [*β* = 0.10, *z =* 1.98, *p* = 0.048, 95% CI (0.02, 4.03)] of desire for power mediation were significant.

However, for the participants with slow life history strategies (1 *SD* above the scale mean), the direct effects of economic uncertainty and fast life history strategy interaction [*β* = −0.28, *z =* −0.27, *p* = 0.79, 95% CI (−7.27, 5.51)] and indirect effects [*β* = −0.07, *z =* −0.92, *p* = 0.36, 95% CI (−1.99, 0.72)] of desire for power mediation were not significant. This indicated that the desire for power partly mediated the interactive effect between economic uncertainty and life history strategy on corrupt intentions for fast life history strategists.

## Discussion

Previous studies have already described different behavioral patterns of two life history strategies (e.g., slow strategists prefer long-term investments, whereas fast strategists prefer short-term ones, Hill et al., 2013; White et al., 2013; Mittal and Griskevicius, 2014). The present study further extended such behavioral differences to corrupt intentions and explored the underlying mechanism. The results indicated that the participants with fast life history strategies would have more desire for power and corrupt intentions when facing economic uncertainty, and the desire for power mediated the interactive effect between economic uncertainty and life history strategy on corrupt intentions for fast life history strategists. The current study examined corruption from a developmental and evolutionary perspective rather than solely relating individuals’ present immorality to their current corrupt intentions and behaviors. These findings open the avenue for investigating corruption from an evolutionary perspective. Specifically, there are several theoretical contributions as followings.

First, it is found that the current environmental uncertainty and life history strategies worked together on individuals’ sociomoral mental processes and behaviors such as corrupt intentions. The Biological Sensitivity to Context (BSC, Boyce and Ellis, 2005) suggests that individuals’ behaviors in their later life may be developmentally adaptable to early-life environmental contexts. Moreover, the Adaptive Calibration Model (ACM, Del Giudice et al., 2011) argues that such adaptation is calibrated by one’s life history strategies. Thus, individuals with different life history strategies might extract different cues from the same environments, influencing their behavioral decisions (Griskevicius et al., 2011a,b; Mittal and Griskevicius, 2014). The sensitization model suggests that economic environmental uncertainty may bring more influence to fast life history strategies than slow life history strategies, resulting in more diversified investment and purchase (White et al., 2013), more risk-taking, approaching temptation faster (Griskevicius et al., 2013), and having children sooner (Griskevicius et al., 2011a). The present study extended such different influences to corrupt intentions by demonstrating that the participants with fast life history strategies were more likely to be aware of the environmental economic uncertainty and thus had more desire for power and corrupt intentions when facing uncertain environments.

Second, we argued that the slow life history strategists were insensitive to environmental uncertainty. The desire for power and corrupt intentions of slow life history strategists did not significantly differ between control and economic uncertainty groups. The ACM suggests that repeated exposures to safe and stable environments lead individuals to hold slow life history strategies (Del Giudice et al., 2011). A plausible interpretation for such insignificance might be that a safe and stable childhood environment shaped their life history strategies as being slow. Consequently, these individuals might exhibit a heightened sensitivity to similar safe and stable environments later in life (Sng et al., 2017), and show less reactions to economic uncertainty. In addition, it is interesting to find that after reading the neutral materials, the participants with slow life history strategies had more desire for power than those with fast life history strategies. This might be because slow life history strategists were more sensitive to safe and stable environments. More desire for power from slow strategists was swayed by their long-term life goals under stable environments, such as enhancing their prestige (Maner and Hasty, 2022; Zhu et al., 2022), rather than obtaining more resources (*cf.* the following discussion about more desire for power of fast life history strategists).

The third facet is that the desire for power mediated the interactive effects between economic uncertainty and life history strategy on corrupt intentions. That is, economic uncertainty led different strategists to diverge in their desire for power and thus influenced their corrupt intentions. This finding was consistent with the previous demonstrations that individuals with life history strategies were likely to use different resource competing strategies (Chen, 2017; Xu, 2021). Though individuals with fast life history strategies are inclined to obtain resources through inappropriate coercive strategies, the cost of traditional ways (e.g., stealing, deception and violence) is large and uneconomical. Thus, fast life history strategists might choose an easier way (i.e., more covert coercive strategies) to acquire resources, such as acquiring power first for more further resources.

Fourth, the current study enriches the literature on corruption from an evolutionary perspective. Previous studies on corruption explored the individual characteristics and environmental factors separately. Some focused on individual characteristics, such as social status (Gu et al., 2020), social dominance orientation (Tan et al., 2016b), and self-esteem (Liang et al., 2016). Others focused on environmental contexts, such as social norms (Köbis et al., 2015) and air pollution (Cole, 2007). The current study adopted a holistic approach to consider corruption, examining the interactive effects between environment (i.e., environmental uncertainty) and individual traits (i.e., life history strategy) on desire for power and corrupt intentions.

Last but not least, the current study has important applications in our daily life. Although life history strategies can be understood as an adaptation to environments under particular circumstances and are neither inherently good nor bad, fast life history strategists might show more norm-violation behaviors such as anti-social behaviors (Figueredo et al., 2004), murder, robbery or assault (Dunkel et al., 2013) and corruption in the present study. However, it is fortunate that such corrupt intentions of fast strategists might controlled by environment certainty to some extent. Although in recent years, the global environment has become more and more uncertain with an increasing number of regional conflicts and wars around the world, we could still adjust the uncertainty of some micro environments (e.g., a company, school or social organization) to reduce corruption conducted by fast strategists. For example, providing a stable company environment such as a sable and achievable promotion prospect and no sudden layoffs might be helpful. Besides, corruption occurs when people in the positions of power or authority abused their influence to violate moral norms for their own benefit (Dungan et al., 2014), which suggests that fast life history strategists in powerful positions of some organizations should be paid more attention. A stable company environment for them might be more helpful.

### Limitations and future directions

Despite the novelty of the current study, several limitations should be noted. First, we only recruited first- or second-year college students. Further studies might use different samples such as officers considering that they have more opportunities to do corrupt practices. Second, the priming materials in the current study including economic decay and difficulty in finding jobs might also trigger intragroup competition anxiety which might also calibrate individuals’ life-history strategies (Zhu et al.,2019). Future research might refine the priming materials (e.g., differing economic uncertainty from decay and only priming economic uncertainty, such as price increases). Last, given the recent criticisms on the ambiguities of the Mini-k questionnaire, it should be cautious in interpreting the current results (Copping et al., 2014; Richardson et al., 2017; Sear, 2020). Although Mini-k measures have been widely used in previous psychological studies on life history strategies and their related behaviors, it is still important to acknowledge the potential accuracy biases in measures that all humans are assumed to lie somewhere on this single continuum of fast or slow life history strategies.

In conclusion, from a life history theory perspective, the current study provides additional evidence for the relationships between life history strategy, economic uncertainty, desire for power, and corrupt intentions. Despite the above limitations, the present study is the first study to understand the mental processes and behaviors of corruption from an evolutionary and developmental perspective and enhances our understanding of the adaptive significance on corruption.

## Data availability statement

The raw data supporting the conclusions of this article will be made available by the authors, without undue reservation.

## Ethics statement

The studies involving humans were approved by School of Social Development and Public Policy Fudan University. The studies were conducted in accordance with the local legislation and institutional requirements. The participants provided their written informed consent to participate in this study. Written informed consent was obtained from the individual(s) for the publication of any potentially identifiable images or data included in this article.

## Author contributions

XS: Writing – review & editing, Writing – original draft, Visualization, Software, Methodology, Investigation, Formal analysis, Data curation. LZ: Writing – review & editing, Writing – original draft, Supervision, Project administration, Funding acquisition, Data curation, Conceptualization.
